# A generic model for individual leaf size in maize, sorghum and pearl millet

**DOI:** 10.1093/aob/mcaf328

**Published:** 2025-12-26

**Authors:** P A Demarco, E J van Oosterom, J Kholová, G L Hammer

**Affiliations:** Queensland Alliance for Agriculture and Food Innovation (QAAFI), The University of Queensland, St Lucia 4072, Australia; ARC Centre of Excellence for Plant Success in Nature and Agriculture, The University of Queensland, St Lucia 4072, Australia; Queensland Alliance for Agriculture and Food Innovation (QAAFI), The University of Queensland, St Lucia 4072, Australia; ARC Centre of Excellence for Plant Success in Nature and Agriculture, The University of Queensland, St Lucia 4072, Australia; Plant Growth Collaborative Research Platform, The University of Queensland, St Lucia 4072, Australia; International Crops Research Institute for the Semiarid Tropics (ICRISAT), Patancheru 502 324, Andhra Pradesh, India; Department of Economics and Development, Faculty of Economics and Management, Czech University of Life Sciences Prague 165 00, Prague, Czech Republic; Queensland Alliance for Agriculture and Food Innovation (QAAFI), The University of Queensland, St Lucia 4072, Australia; ARC Centre of Excellence for Plant Success in Nature and Agriculture, The University of Queensland, St Lucia 4072, Australia

**Keywords:** Canopy architecture, blade length, blade width, modelling, cereals, leaf area

## Abstract

**Background and Aims:**

Crop growth models (CGMs) are a valuable tool for predicting crop performance in contrasting growing conditions and interpreting crop responses to future scenarios. Inaccuracies in the simulation of leaf area dynamics directly impact estimates of intercepted radiation, biomass production and transpiration demand by the crop, especially during the early stages when the canopy is not yet fully covering the soil. An empirical bell-shaped function of individual leaf area versus leaf position, combined with the response of leaf appearance to thermal time, is used in many CGMs to simulate total leaf area per axis and generate canopy leaf area index. This study proposes that an individual leaf area approach, based on predicting blade length and blade width of successive leaves, can make modelling of leaf area dynamics less empirical, while offering the flexibility to better simulate genotypic, and genotypic × environment interaction effects in sorghum [*Sorghum bicolor* (L.) Moench], maize (*Zea mays* L.) and pearl millet (*Pennisteum americanum* L.).

**Methods:**

A generic model of leaf area by leaf position was developed using data on individual blade length and width compiled from numerous experiments over the period 1990–2022 that involved a broad range of genotypes of sorghum, maize and pearl millet.

**Key Results:**

This study developed and tested a generic individual leaf size model for maize, sorghum and pearl millet, based on relationships quantifying length and width of successive leaves. Generic parameters of an expolinear-logistic model obtained across species and related to total leaf number, as appropriate, facilitated satisfactory predictive performance for blade length, width and leaf area profiles. Genotype-specific parameters improved model predictions in this study.

**Conclusions:**

Improvements in the parameterization of canopy development in CGMs can enhance predictions of genotype × environment × management (G × E × M) interactions to support identifying breeding targets for enhanced yield and strategies for sustainable crop management.

## INTRODUCTION

Crop growth models (CGMs) can be used to study and interpret crop responses across environments by predicting the consequences of modifications on crop performance. Models predict trajectories of plant growth and development through the crop life cycle capturing the dynamic effects of crop trait and management options (Hammer *et al*., 2016). CGMs are utilized to predict genotype ×environment × management (G × E × M) interactions for agronomy and breeding by characterizing environments for breeding trials and capturing the crop physiological mechanisms underlying the dynamics of crop growth and development associated with trait and management manipulations (Copper and Hammer 1996; Chapman *et al*., 2000; Hammer *et al*., 2016).

Complex G × E × M interactions can impact canopy development. Genotypic differences have been reported for canopy size and stay green for sorghum, maize and millet (Maddonni and Otegui, 1996; Borrell *et al*., 1999, 2014; Borrell and Hammer, 2000; van Oosterom *et al*., 2001b; Jordan *et al*., 2012; Kholová *et al*., 2014; Incognito *et al*., 2022). Differences in blade width among sorghum genotypes have been associated with differences in transpiration efficiency (TE) (Zhi *et al*., 2022*a*). Management practices such as sowing density and row spacing will affect crop leaf area development and, hence, canopy size and resource capture dynamics during the crop life cycle (Carberry *et al*., 1985; Maddonni *et al*., 2001; van Oosterom *et al*., 2001*a*, *b*; Blumenthal *et al*., 2003; Whish *et al*., 2005).

Canopy development is characterized by the nature of leaf area production and senescence occurring from emergence to physiological maturity of a crop. Biomass production is associated directly with light interception by the canopy (Hammer *et al*., 2010). Hence, the dynamics of crop leaf area play a key role in crop growth, photosynthesis and transpiration (Muchow and Carberry, 1989; Muchow *et al*., 1990). Leaf area development is a result of leaf appearance and leaf expansion rates, which are affected by temperature and photoperiod (Chenu *et al*., 2008; Ravi Kumar *et al*., 2009). Leaf area determines not only the amount of leaf surface exposed to solar radiation for photosynthesis, but also the amount of transpiring surface of the crop and, thus, TE (Hammer *et al*., 1997). Consequently, inaccuracies in the simulation of leaf area dynamics will directly impact estimates of intercepted radiation, biomass production and transpiration demand by the crop, especially during the early stages when the canopy is not yet fully covering the soil. This can adversely affect CGM performance in predicting crop growth and yield.

Similar individual leaf area profiles along the main culm, represented as a bell-shaped function in relation to leaf position, have been reported for maize, sorghum and pearl millet (Hammer *et al*., 1987; Keating and Wafula, 1992; Carberry *et al*., 1993; van Oosterom *et al*., 2001*a*; Chenu *et al*., 2008). It has been demonstrated that area of the largest leaf depends on the total number of leaves on the culm (Warrington and Kanemasu, 1983*b*; Muchow and Carberry, 1990; van Oosterom *et al*., 2001*a*). The total number of leaves varies with genotype (maturity) and environmental factors such as photoperiod, which can affect leaf number through affecting the duration of the vegetative phase, and temperature, which can affect leaf number if the rates of development and leaf initiation differ on their base temperatures (Warrington and Kanemasu, 1983*b*; Muchow and Carberry, 1990; Giauffret *et al*., 1995; Ravi Kumar *et al*., 2009; Tirfessa *et al*., 2023). Leaf initiation and leaf appearance in cereals show a linear relationship with temperature, which can be affected by photoperiod (Warrington and Kanemasu, 1983*a*; Lafarge and Tardieu, 2002). Blade length and blade width also present similar size profiles along the main culm for maize, sorghum and pearl millet (Birch *et al*., 1997; van Oosterom *et al*., 2001*a*; Chenu *et al*., 2008). Blade width has been associated with genotypic differences in sorghum and maize (Maddonni *et al*., 2001; Kim *et al*., 2010*a*; Zhi *et al*., 2022*a*, *b*), whereas blade length was associated with environmental effects (Kim *et al*., 2010*b*). The similarities in patterns of leaf area development across maize, sorghum and pearl millet suggest that a common generic approach to modelling blade width, blade length and leaf area is possible across species.

Canopy development in the sorghum crop model in the Agricultural Production Systems Simulator (APSIM) has been predicted on a whole plant basis through a relationship between total plant leaf area (TPLA) and thermal time (Hammer *et al*., 2016). TPLA combines the number of fully expanded leaves, their individual size and tiller number, and includes an adjustment for the area of expanding leaves (Hammer *et al*., 1993, 2010). This methodology has descriptive parameters that capture the shape of the canopy development function and depend on a user-defined tiller number per plant. In recent modifications associated with predicting tiller dynamics (Hammer *et al*., 2023), an individual leaf approach has been adopted (Dwyer and Stewart, 1986; Carberry *et al*., 1993). In essence, TPLA becomes an integration of the area of individual leaves, which can be represented by a bell-shaped function that calculates the area of individual leaves as a function of their position and the total leaf number (TLN) of an axis. Parameters of the leaf area profile bell-shaped function are related to TLN (Hammer *et al*., 2023). The number of fully expanded leaves at any time is calculated as the product of thermal time elapsed since emergence, and the leaf appearance rate (Chenu *et al*., 2008). Actual crop leaf area is the product of plant density and leaf area per plant. Green leaf area index is the difference between the total plant leaf area and the senesced leaf area (Hammer *et al*., 2010). van Oosterom *et al*. (2001*b*) suggest that the individual leaf area approach of modelling crop leaf area offers the flexibility to simulate genotypic differences in leaf area index and tillering, which can result in genotype-by-environment interactions for panicle number.

An individual leaf area approach, based on blade length and width of successive leaves, could potentially make modelling of leaf area more mechanistic, thus offering the flexibility to simulate genotypic and genotypic × environment differences more realistically. Another potential use of dissecting leaf size into leaf width and length is the opportunity to incorporate associations with relevant traits into CGMs, such as leaf blade width with TE. This could open avenues to better incorporate traits related to canopy development into breeding. Here we develop and test a generic individual leaf area model for maize, sorghum and pearl millet, based on relationships quantifying blade length and blade width of successive main shoot leaves. A comprehensive dataset from prior experiments was compiled for model fitting and independent experiments were conducted to generate data for model testing.

## MATERIALS AND METHODS

### Model framework and parameterization

**Fig. 1.**
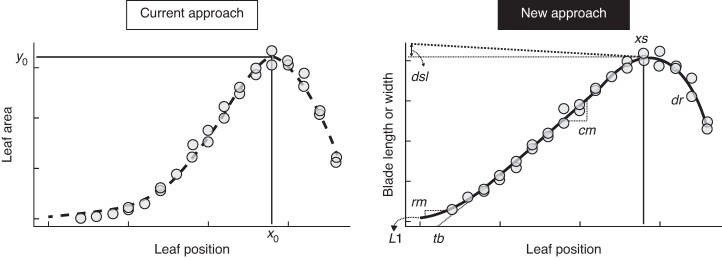
Schematic diagrams of the current approach for fitting leaf area versus leaf position and a new approach for fitting blade length or width versus leaf position. Circles represent a sorghum genotype with TLN of 18.

The current approach to calculating leaf area in the APSIM-sorghum model (Fig. 1) consists of a bell-shaped function (APSIM v.7.10 and APSIM Next Generation – www.apsim.info) that calculates the area of individual leaves as a function of the TLN of the axis (Carberry *et al*., 1993):


(1) $$Y=Y_{0}\;exp(a(x-x_{0}{)}^{2}+\;b(x-x_{o}{)}^{3})$$

where *X*_0_ is the position of the largest leaf, *Y*_0_ is the mature area of the largest leaf, *a* is an empirical constant determining the breadth of the bell-shaped curve and *b* an empirical constant determining the skewness of the bell-shaped curve.

The bell-shaped function for leaf size distribution has been utilized to represent leaf area in crops including the three crops in this study (Muchow and Carberry, 1989; Carberry *et al*., 1993; van Oosterom *et al*., 2001*a*). However, Hammer *et al*. (2023) and Diancoumba *et al*. (2024) reported for sorghum that the empirical coefficients of the leaf size distribution function (i.e. ‘*a*’ and ‘*b*’) were stable only for TLN between 10 and 20.

The proposed model predicts the blade length and width of each leaf on the main culm of a plant (Fig. 1). The model combines an initial expolinear term with a subsequent logistic decay term to form a three-phase expolinear-logistic model, similar to that proposed for kernel size by Fernández *et al*. (2022). It is applied to either blade length or width (mm) where *x* is the leaf position (leaf number) on the plant from the bottom to the top (Fig. 1):

expolinear term:


(2) $$\mathrm{Leaf}\;\mathrm{length}\;\mathrm{or}\;\mathrm{width}=L1+\left(\frac{cm}{rm}\right)\;ln(1+exp^{rm(x-tb-1)})\;for\;x<xs,$$

logistic term:


(3) $$\mathrm{Leaf}\;\mathrm{length}\;\mathrm{or}\;\mathrm{width}=\frac{Y_{\max}\times Ys_{\mathrm{level}}}{Y_{\max}+\;(Ys_{\mathrm{level}}-Y_{\max})\;exp^{\left(dr(X-xs)\right)}}\;for\;x\geq xs,$$

where $Y_{\max}=L1+(\frac{cm}{rm})\;ln(1+\;exp^{rm(xs-tb-1)})$, *L*1 is the blade length or width for the first leaf (coleoptile leaf), *cm* is maximum slope (absolute increase in length or width of successive leaves) during the linear phase (mm per leaf), *rm* is the maximum increase of maximum length or width of consecutive leaves during the exponential phase, *tb* is leaf position where the extrapolated *cm* slope crosses the *x*-axis, *xs* is leaf position at which the maximum *Y* (length or width) is achieved, $Ys_{\mathrm{level}}=Y_{\max}+\;dsl$, where *dsl* is the difference between the maximum *Y* at *xs* (*Y*_max_) and the upper horizontal asymptote of the logistic decay phase, and *dr* is the rate of decay of the logistic phase (Fig. 1).

Leaf area is then calculated by multiplying leaf length, leaf width and a leaf shape factor:


(4) Leafarea=bladelength×bladewidth×leafshapefactor

The leaf shape factor used for all leaves of each crop was 0.71, except for the flag leaf where the shape factor used was 0.635 (van Oosterom *et al*., 2021).

### Model fitting

The generic model of individual leaf size by leaf position was fitted using as a training dataset of length and width data from individual leaves compiled from multiple experiments from 1990 to 2014 involving a broad range of sorghum, maize and pearl millet genotypes (Table 1). Experiments across multiple locations and years were used to describe individual blade length, width and TLN of different maize, sorghum and pearl millet genotypes across different plant densities (Table 1). Blade length was measured on fully expanded leaves (leaves with ligule exposed) from the ligule to the tip of the leaf with a ruler or measuring tape. Blade width was recorded on fully expanded leaves for the widest section of the leaf using a ruler or caliper. Only data from leaves on the main culm were used and no data from tillers were included. In this analysis, datasets were combined to estimate species-level parameters for the individual blade length and width models.

**Table 1.** Details of experiments contributing to the leaf size dataset indicating the number of genotypes and total number of observations for blade length and width (*n*) used in the analysis.

Species	ID	Location	Year	Experiment	Genotype	pl_m2	*n*	Total leaf number		Blade length (mm)				Blade width (mm)				References
								Min.	Max.	Mean	s.d.	Min.	Max.	Mean	s.d.	Min.	Max.	
Sorghum	1	Gatton, Australia	2006	Field	7	7.48	2262	16	21	435.03	243.53	10	900	54.38	30.41	5	113	van Oosterom *et al*. (2011)
	2	Gatton, Australia	2007	Solar-weave enclosure	9	Not reported	469	15	19	482.15	250.85	13	850	56.07	30.27	6	101	van Oosterom *et al*. (2011)
	3	Gatton, Australia	2007	Solar-weave enclosure	2	Not reported	101	16	18	431.19	262.58	6	854	54.44	33.41	5	105	van Oosterom *et al*. (2011)
	4	Gatton, Australia	2011	Solar-weave enclosure	20	Not reported	1116	9	18	417.56	235.59	7	859	46.67	30.4	5	118	van Oosterom *et al*. (2021)
	5	Gatton, Australia	2011	Solar-weave enclosure	21	Not reported	1244	9	20	418.94	258.68	13	966	47.77	34.87	4	131	van Oosterom *et al*. (2021)
	6	Gatton, Australia	1990	Field	1	16	310	15	16	434.2	274.77	14	865	45.74	29.51	3	90	Muchow and Sinclair (1994)
	7	Gatton, Australia	1990	Field	1	16	337	15	19	459.16	269.96	12	860	50.99	29.14	4	93	Muchow and Sinclair (1994)
	8	Gatton, Australia	1991	Field	1	16	319	15	18	465.8	290.13	12	936	50.73	31.63	4	101	Muchow and Sinclair (1994)
	9	Gatton, Australia	1998	Field	1	7.39	1877	14	18	454.46	253.23	15	880	56.9	32.85	5	117	Lafarge *et al*. (2002)
	10	Patancheru, India	2014	Field	3	Not reported	2911	19	26	617.89	314.35	50	1230	62.69	30.43	3	135	J. Kholová (unpublished)
	11	Sotuba, Mali	1996	Field	3	Not reported	1831	23	44	625.75	315.06	10	1135	74.88	38.44	4	139	J. Kholová (unpublished)
	12	Sotuba, Mali	1996	Field	3	Not reported	1475	19	36	556.89	295.01	10	1056	68.36	39.58	3	146	J. Kholová (unpublished)
	13	Warwick, Australia	2005	Solar-weave enclosure	2	Not reported	92	14	16	384.38	223.8	12	710	46.21	26.31	6	83.5	van Oosterom *et al*. (2011)
Maize	14	Not reported	Not reported	Field	12	Not reported	3090	11	25	591.49	299.75	10	1210	65.29	34.16	6	136	S. C. Chapman (unpublished)
	15	Gatton, Australia	2006	Solar-weave enclosure	2	Not reported	138	16	18	560.56	275.82	16	921	75.98	43.12	9	136	van Oosterom *et al*. (2016)
	16	Gatton, Australia	2006	Solar-weave enclosure	2	Not reported	100	16	18	525.46	257.84	26	1000	68.31	38.65	8	124	van Oosterom *et al*. (2016)
	17	Gatton, Australia	2007	Solar-weave enclosure	2	Not reported	84	16	17	546.87	272.66	30	896	68.68	37.04	12	123	van Oosterom *et al*. (2016)
	18	Gatton, Australia	2008	Solar-weave enclosure	6	Not reported	431	17	19	587.81	269.36	10	969	76.31	39.75	15.5	137	van Oosterom *et al*. (2016)
	19	Gatton, Australia	2011	Solar-weave enclosure	8	Not reported	536	16	18	597.03	280.09	33	989	73.98	40.66	8	137	van Oosterom *et al*. (2016, 2021)
	20	Gatton, Australia	2011	Solar-weave enclosure	8	Not reported	580	17	19	596.99	287.15	25	1039	75.56	40.22	4	137	van Oosterom *et al*. (2016, 2021)
	21	Gatton, Australia	2012	Solar-weave enclosure	7	Not reported	1053	18	20	600.25	252.64	44	938	77.39	35.85	14	131	E. J. van Oosterom (unpublished)
	22	Gatton, Australia	1990	Field	1	7	354	17	19	539.52	236.15	41	855	77.84	36.26	6	131	Muchow and Sinclair (1994)
	23	Gatton, Australia	1990	Field	1	7	381	18	21	537.5	252.31	38	910	75.06	34.81	12	128	Muchow and Sinclair (1994)
	24	Gatton, Australia	1991	Field	1	7	368	17	20	444.36	183.14	30	772	79.81	37.74	11	133	Muchow and Sinclair (1994)
	25	Woodland, USA	2010	Field	18	Not reported	1058	19	22	674.12	265.89	80	1067	74.7	26.42	15	124	E. J. van Oosterom (unpublished)
Pearl millet	26	Jodhpur, India	1996	Field	1	11.1	460	15	24	480.99	262.1	23	1110	21.89	11.73	3	49	van Oosterom *et al*. (2001*a*, *b*)
	27	Patancheru, India	1996	Field	4	9.29	780	14	20	385.61	213.18	21	836	22.29	12.18	3	51.5	van Oosterom *et al*. (2001*a*, *b*)
	28	Patancheru, India	1997	Field	4	2.5	736	13	27	486.28	253.16	24	1004	28.21	16.15	2.5	70.5	van Oosterom *et al*. (2001*a*, *b*)
	29	Patancheru, India	1996	Field	4	11.89	1261	11	20	365.99	212.51	18	855	21.85	12.61	2	59	van Oosterom *et al*. (2001*a*, *b*)

To account for both genotype and environmental effects on phenology across experimental set-ups, observations were first grouped by the total number of leaves within genotypes and species (hereafter referred to as ‘TLN groups’). The expolinear-logistic function was fitted using the non-linear least squares model (nlsLM) from the *minpack.lm* package (Elzhov *et al*., 2023) in R studio to data on blade length and width versus leaf position. The helper function nlsLMList from the *nlraa* package (Miguez, 2023) was used to facilitate model fitting across groups. Parameter values and standard errors from the expolinear-logistic model fits were obtained for each group.

The association between TLN and the obtained parameters was tested for each species. A linear relationship was fitted to the position of the largest leaf (parameter *xs*) and TLN for maize and pearl millet. For sorghum, a segmented regression was fitted for this relationship using non-linear models. A bilinear function was fitted to the decay of the logistic phase (parameter *dr*) and TLN using non-linear models. Outliers for *dr* were removed based on their bivariate distribution quantile. These were usually associated with low TLN plants.

## RESULTS

### Relationship of model parameters with total leaf number

The association of fitted model parameters with TLN was tested for all species, considering genotype within species as a random effect (Table 2). The association with TLN was consistently significant across species for both blade length and width for the parameters *xs* (leaf position of maximum leaf length or width) and *dr* (rate of decay of the logistic phase). Therefore, these two parameters were considered as defined by the total number of leaves in the modelling approach proposed in this study. The remaining parameters were set as fixed across all TLN values, using the average value fitted within each TLN group for each species.

**Table 2.** Level of significance of association of each parameter with total leaf number for blade length and width across maize, sorghum and pearl millet; *cm*, *rm*, *tb*, *xs*, *dr*, and *dsl* are parameters of the three-phase expolinear-logistic model (see Fig. 1 for details).

Parameter	Maize		Sorghum		Pearl millet	
	Leaf length (mm)	Leaf width (mm)	Leaf length (mm)	Leaf width (mm)	Leaf length (mm)	Leaf width (mm)
*cm*	ns	*	ns	ns	ns	ns
*rm*	***	ns	ns	ns	ns	ns
*tb*	***	**	*	ns	ns	ns
*xs*	***	***	***	***	***	***
*dr*	***	***	**	***	**	*
*dsl*	Ns	ns	*	ns	*	*

ns: not significant; asterisks indicate significant at *P < 0.05, **P < 0.01, ***P < 0.001.

**Fig. 2.**
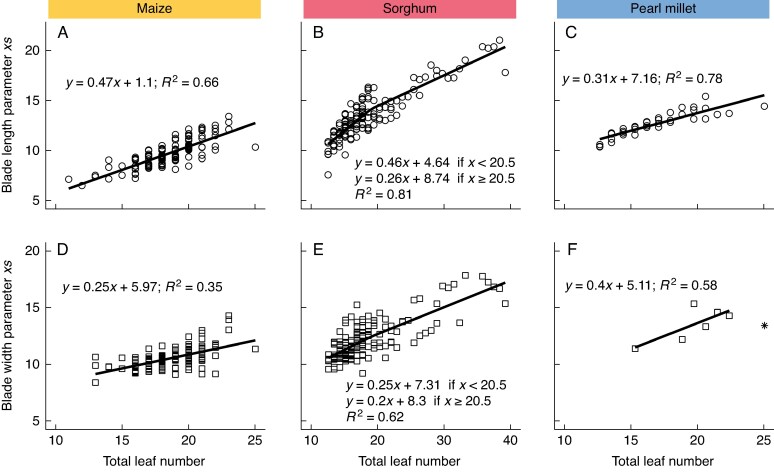
Relationship between position of the largest leaf (parameter *xs*) and total leaf number for maize blade length (A) and width (D), sorghum blade length (B) and width (E), and pearl millet blade length (C) and width (F). Across crops, blade length is represented by open circles, and blade width by open squares. Asterisk indicates outlier excluded from the regression line for pearl millet. Colours represent species: yellow for maize, red for sorghum and blue for pearl millet.

As TLN increases, the position of the largest leaf increases linearly for blade length and blade width across crops (Fig. 2). These results are consistent with previous studies for sorghum and pearl millet that demonstrated this linear relationship (Keating and Wafula, 1992; Carberry *et al*., 1993; van Oosterom *et al,* 2001*a*). However, due to the inclusion of sorghum genotypes in this study with much larger number of leaves than in the previous studies, a segmented regression was required for the relationship of blade length and width on TLN. The break in the segmented regression was defined at a TLN of 20.5. Hence, the majority of genotypes with TLN < 21 will be represented using one slope, whereas genotypes with larger leaf numbers will require the second slope of the segmented relationship.

**Fig. 3.**
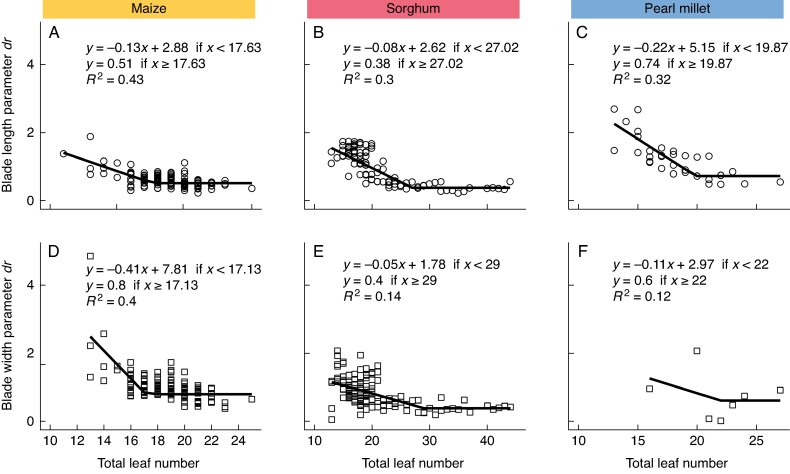
Relationship between rate of decay in the logistic phase (parameter *dr*) and total leaf number for maize blade length (mm) (A) and width (mm) (D), sorghum blade length (B) and width (E), and pearl millet blade length (C) and width (F). Across crops, blade length is represented by open circles, and blade width by open squares. Colours represent species: yellow for maize, red for sorghum and blue for pearl millet.

The decay of the logistic phase represents the portion of the curve from the position of the largest leaf up to the flag leaf. The rate of decay of the logistic phase (*dr*) decreases as the TLN increases for blade length and blade width in all species until a certain number of leaves, after which the rate remains constant (Fig. 3). The results suggest that the exponential rate of decay in leaf length or width for genotypes with low TLN was greater than for genotypes with larger TLN. However, for all crops, above a certain TLN, the rate of decay becomes constant. For maize, the rate decreases until a TLN of 18 for blade length and a TLN of 17 for blade width (Fig. 3A, D). In sorghum, the rate decreases until a TLN of 27 for blade length and a TLN of 29 for blade width (Fig. 3B, E), and for pearl millet the rate decreases until a TLN of 20 for blade length and a TLN of 22 for blade width (Fig. 3C, F).

The complete sets of generic model parameters fitted for blade length and width for each species are given in Table 3. For length and width parameters *cm*, *tb*, *rm* and *dsl*, the mean by species was used for the generic model. For parameters *xs* and *dr*, the parameter value for each species was based on the associations with TLN shown in Figs 2 and 3.

**Table 3.** Model parameter values and their standard errors (in parentheses) for maize, sorghum and pearl millet. Parameter *cm* is the maximum absolute slope during the linear phase, *rm* is maximum increase of maximum length or width of consecutive leaves during the exponential phase, *tb* is leaf position where the extrapolated *cm* slope crosses the *x*-axis. *xs.*a and *xs.*b are the intercept and slope of the linear relationship between *xs* versus TLN for maize and pearl millet; for sorghum a segmented regression was fitted and *xs.*a represents the intercept, *xs.*b the slope from 1 up to 20.5 total leaf number, which is the break point (*xs*.bp), and *xs*.c represents the slope for above 20.5 total number of leaves. *dr*.a and *dr*.b are the intercept and slope of the relationship of *dr* (rate of decay of the logistic phase) versus TLN, and *dr*.xs represents the breakpoint of this relationship. *dsl* is the delta between the maximum *Y* at *xs* and the upper limit of *Y* used in the calculation of the logistic phase, and *L*1 is the size of the first leaf. Values for blade length and blade width represent the mean ± the standard error in parentheses.

Parameter	Maize		Sorghum		Pearl millet	
	Blade length (mm)	Blade width (mm)	Blade length (mm)	Blade width (mm)	Blade length (mm)	Blade width (mm)
*cm*	123.84 (±3.1)	19.37 (±1.7)	70.5 (±1.45)	11.72 (±0.21)	62.69 (±3.14)	14.99 (±3.14)
*rm*	1.17 (±0.12)	0.58 (±0.05)	1.56 (±0.15)	0.61 (±0.02)	0.92 (±0.12)	0.19 (±0.02)
*tb*	2.02 (±1.14)	4.10 (±0.40)	1.92 (±0.17)	4.04 (±0.15)	1.53 (±0.28)	14.79 (±1.69)
*xs*.a	1.10 (±0.55)	5.97 (±0.55)	4.64 (±0.72)	7.31 (±0.76)	7.16 (±0.5)	5.11 (±3.63)
*xs*.b	0.47 (±0.03)	0.25 (±0.03)	0.46 (±0.04)	0.25 (±0.04)	0.31 (±0.03)	0.40 (±0.17)
*xs*.c	–	–	0.26 (±0.02)	0.20 (±0.02)	–	–
*xs*.bp	–	–	20.50	20.50	–	–
*dr*.a	2.88 (±0.30)	7.81 (±0.8)	2.62 (±0.2)	1.78 (±0.18)	5.15 (±0.71)	2.97 (±4.04)
*dr*.b	−0.13 (±0.02)	−0.41 (±0.05)	−0.08 (±0.01)	−0.05 (±0.01)	−0.22 (±0.04)	−0.11 (±0.21)
*dr*.xs	17.63(±0.34)	17.13 (±0.22)	27.02 (±1.36)	29.00 (±2.9)	19.87 (±0.9)	22.00 (±8.13)
*dsl*	12.53 (±1.49)	0.20 (±0.04)	3.50 (±0.70)	0.18 (±0.03)	1.37 (±0.41)	0.08 (±0.02)
*L*1	51.18 (±0.79)	15.98 (±0.06)	17.59 (±0.79)	4.20 (±0.06)	34.51 (±0.79)	7.06 (±0.06)

### Model performance

**Fig. 4.**
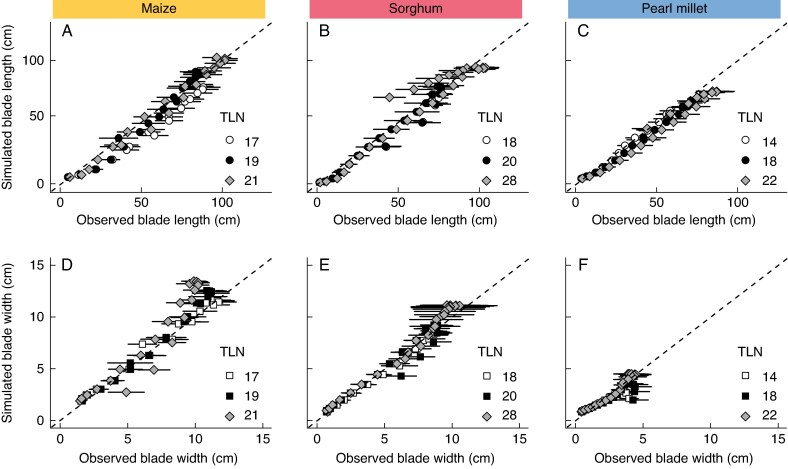
Simulated versus observed blade length (cm) for a range in total leaf number (TLN) for maize (A), sorghum (B) and pearl millet (C) and simulated versus observed blade width (cm) for maize (D), sorghum (E) and pearl millet (F). Points indicate the mean by the position of the leaf on the plant, and bars indicate the standard deviation of the mean. Different shapes represent the TLN of the crop. The dashed line represents the 1:1 line. Colours represent species: yellow for maize, red for sorghum and blue for pearl millet.

Model performance was evaluated based on the goodness-of-fit between model predictions and observed training data, revealing satisfactory accuracy in simulating blade length and width across species. The generic models showed strong agreement with observed values across a range of species × TLN combinations (Fig. 4). For maize, groups with TLN of 17, 19 or 21, which is common for this crop, aligned well with the 1:1 line, indicating accurate simulation of blade length and width. However, the spread associated with the standard deviation of the mean in all groups suggested that calibration by individual genotypes will be needed to obtain a more accurate simulation of leaf size when using this model. Similar results were obtained for sorghum and pearl millet. In sorghum, the group with the larger TLN demonstrated greater variability. This could be related to fewer genotypes and experimental samples in this group. However, the results demonstrate overall that the use of generic parameters by species in the model can satisfactorily simulate genotypes with a wide range of TLN.

### Model testing

A compilation of data from glasshouse experiments conducted during 2022 and 2023 at the Gatton campus, Queensland (27°33′15.0″S, 152°20′21.8″E) was used as an independent dataset to test the expolinear-logistic model. The experiments included detailed measurements of blade length and width for two sorghum genotypes, one pearl millet cultivar and one maize hybrid. In both experiments, seeds were sown in pots and genotypes were arranged in a randomized complete block design (RCBD) with plant densities of 1 and 7 m^2^ for experiments conducted in 2022 and 2023, respectively. Pots were uniformly irrigated, and no nutrient limitations were observed. Sorghum inbred lines were Tx7000 and R931945-2-2, the latter an elite germplasm line developed by the Queensland Department of Primary Industries (QDPI) sorghum breeding programme (Jordan *et al*., 2012; George-Jaeggli *et al*., 2013). Sorghum seeds were provided by the UQ-GRDC-QDPI sorghum pre-breeding programme. One commercial maize hybrid and one pearl millet cultivar were included to represent extremes in leaf size. Sorghum genotypes were selected based on previous studies where blade width was reported to be associated with TE of the crop (Zhi *et al*., 2022*a*, *b*). The size of individual fully expanded leaves was determined by non-destructively measuring blade width and length of all leaves on the main culm. Blade width was measured on the widest section of the leaves with a Vernier caliper. Blade length was measured with a ruler from the ligule to the tip.

**Fig. 5.**
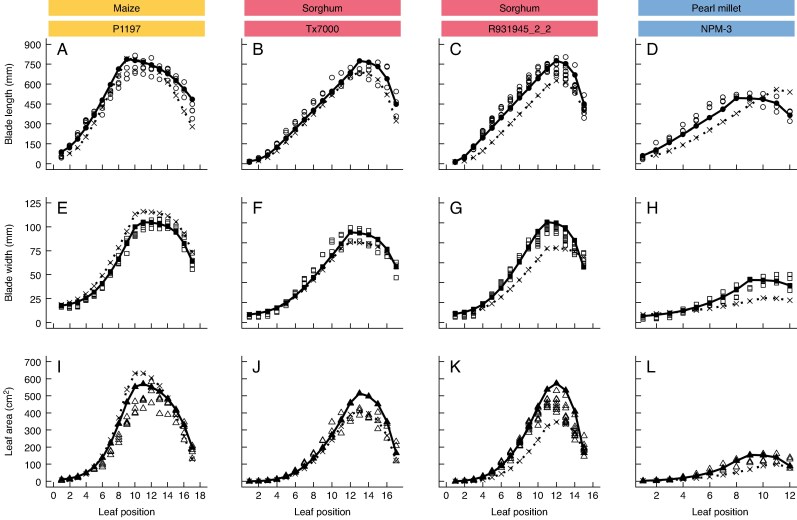
Blade length, width and leaf area versus leaf position for maize P1197 (A, E, I), sorghum Tx7000 (B, F, J), sorghum R931945_2_2 (C, G, K) and pearl millet NPM-3 (D, H, L), respectively. Circles represent blade length, squares represent blade width and triangles represent leaf area. Open black symbols represent data from the testing dataset; model predictions with genotype-specific parameters are represented by solid lines and closed symbols. Predictions with generic parameters are represented by dotted lines and cross symbols. Colours represent species: yellow for maize, red for sorghum and blue for pearl millet.

The expolinear-logistic model was fitted to the testing data for each genotype (Supplementary Data Table S1). The fitted parameters for one maize, two sorghum and one pearl millet genotype were used to simulate blade length, width and leaf area. Each aspect of leaf size was accurately predicted for the four genotypes (Fig. 5). For blade length, the position of the largest leaf occurred at a lower leaf position in the maize genotype with 17 TLN (Fig. 5A) compared to a sorghum genotype with the same TLN (Fig. 5B), probably due to the position of the ear in the maize plants. There was little difference in the size of the flag leaf for blade length, width and area among genotypes and species. While the generic model had been demonstrated to accurately predict blade length and width using species-generic parameters (Fig. 4), calibration of model parameters for the specific genotypes in this test improved model predictions (Fig. 5; Table S2). Model fit to the independent testing dataset was evaluated for generic versus specific parameters for the predictions of blade length, width and leaf area using root mean square error (RMSE) and the coefficient of determination (*R*^2^). These results indicated that while the form of the generic expolinear-logistic model was appropriate, genotypic parameterization could improve accuracy of the leaf size predictions.

## DISCUSSION

The results of this study demonstrate that across the C4 species maize, sorghum and pearl millet, it is possible to model individual blade length, width and leaf area of successive main shoot leaves with a generic expolinear-logistic function. This generic approach to modelling leaf dimensions across species reflects similar findings for the area of individual leaves in relation to leaf position reported for each of sorghum, maize and pearl millet (Hammer *et al*., 1987; Keating and Wafula, 1992; Carberry *et al*., 1993; Van Oosterom *et al*., 2001*b*; Chenu *et al*., 2008). van Oosterom *et al*. (2001*a*) describe the parameterization and validation of leaf area profiles in pearl millet and the approach has been used successfully to simulate leaf area profiles in sorghum and maize (Carberry and Abrecht, 1991; Keating and Wafula, 1992). This suggests common control mechanisms across maize, sorghum and pearl millet.

By incorporating the expolinear term, the new approach better captures the size of the smaller leaves compared to the bell-shaped function (Fig. 3; Hammer *et al*., 2023) when the canopy still does not fully cover the soil. This advantage is relevant when simulating canopy size at the early stages of the crop and opens opportunities to provide insights into genotypic effects on consequential traits for canopy dynamics such as tillering (Kim *et al*., 2010*a*). Additionally, the current bell-shaped approach does not simulate blade length and width for leaves on the plant. In the new expolinear-logistic function, blade length and blade width are calculated in order to then calculate leaf area, providing the opportunity to link genotypic differences in blade length or width to related traits, such as TE (Zhi *et al*., 2022*a*, *b*).

While the generic model approach reported here does not focus on genotype-specific parameters, the results of tests on the independent dataset (Fig. 5) highlight the possibility of parameterizing for specific genotypes with this model. Using the expolinear-logistic model to simulate blade length and width in CGMs will open possibilities to explore the potential consequences of genotypic variation in leaf size profiles. It has been shown that genotypic differences exist in leaf size distribution for sorghum, maize and pearl millet, particularly in relation to blade width (Maddonni *et al*., 2001; Kim *et al*., 2010*a*). Relevant traits for crop improvement, such as TE, have been related to aspects of canopy architecture, such as leaf width (Zhi *et al*., 2022*a*, *b*), but such connections are not incorporated in most CGMs.

Genotypic variation in leaf size profiles has also been associated with variation in tillering. Kim *et al*. (2010*a*) and Alam *et al*. (2014*a*, *b*) demonstrated that genetic variation in tillering for sorghum was related to effects on source–sink balance, which was related to leaf size. Accurate predictions of leaf size during early vegetative stages can provide insights on genotypic variation in tillering dynamics in sorghum (Hammer *et al*., 2023). In this study, the results also show the differences in blade width across species (Fig. 5E–H). The wider leaves in maize and narrower leaves in pearl millet are consistent with their lower (or higher) propensity to tiller. A more generic approach to modelling leaf size profiles and tillering has the potential to capture these intra- and inter-species differences in tillering as emergent consequences of differences in leaf area profiles. Although our results demonstrate the added value of explicitly modelling blade width and length, quantifying the resulting prediction accuracy of gains in canopy and tillering dynamics relative to the current approach remains a critical next step for future research.

A new approach to using CGMs has been described by Messina *et al*. (2018) and Hammer *et al*. (2019), whereby using mechanistic models to link objectives of plant breeding and quantitative genetics with important plant and agronomic traits can contribute to enhancing grain yield potential and yield stability. This demonstrates the progression from gene-to-phenotype (G2P) models of quantitative genetics to applications using mechanistic crop models (CGM-G2P), which can provide integrated strategies for breeding programmes based on trait genetics, crop physiology, breeding and agronomy (Cooper *et al*., 2021). This study offers the possibility to incorporate relevant canopy architecture insights into the CGM-G2P pipeline to better investigate canopy structure as an option for *in silico* studies of plant breeding strategies.

## CONCLUSIONS

This study developed and tested a generic individual leaf size model for maize, sorghum and pearl millet, based on relationships quantifying the length and width of successive leaf positions. The model was parameterized using a comprehensive dataset (1990–2022) encompassing diverse genotypes and environmental conditions. Generic parameters of an expolinear-logistic model obtained across species, and related to TLN as appropriate, facilitated satisfactory predictive performance for blade length, width and leaf area profiles. Detailed model testing on independent data revealed that genotype-specific parameters improved model predictions. The approach developed can make modelling of leaf area dynamics less empirical, thus potentially offering the flexibility to simulate genotypic and genotype × environment effects. The present study can enhance CGM parameterization of canopy architecture across genotype × environment × management (G × E × M) combinations, guiding the way forward to concrete strategies for identifying future target traits that optimize light capture, transpiration efficiency and tillering under contrasting environments and management conditions.

## Supplementary Material

mcaf328_Supplementary_Data
