# Supplementary material for: A generic model for individual leaf size in maize, sorghum and pearl millet
Source: Ann Bot. 2025 Dec 26;137(4):920–30. doi: 10.1093/aob/mcaf328 (PMC13095885; doi:10.1093/aob/mcaf328)
Supplement: mcaf328_Supplementary_Data [file mcaf328_supplementary_data.docx]

**[Supplementary Information]**

Table S1: Fitted parameter values for one maize, two sorghum and one pearl millet genotype used to simulate blade length, width and leaf area in the independent model testing. Parameters are as defined in Table 3.

| Parameter | Maize P1197 | | Sorghum Tx7000 | | Sorghum R931945_2_2 | | Pearl millet NPM-3 | |
| --- | --- | --- | --- | --- | --- | --- | --- | --- |
|  | Leaf length (mm) | Leaf width (mm) | Leaf length (mm) | Leaf width (mm) | Leaf length (mm) | Leaf width (mm) | Leaf length (mm) | Leaf width (mm) |
| cm | 120 | 19.37 | 70.5 | 12.50 | 73.90 | 17.50 | 62.69 | 14.99 |
| rm | 0.8 | 0.58 | 0.50 | 0.61 | 3.96 | 0.61 | 1.50 | 0.40 |
| tb | 1.5 | 4.80 | 1.50 | 4.04 | 0.5 | 4.04 | 0.03 | 6.50 |
| xs.a | 1.13 | 5.97 | 4.64 | 7.31 | 4.64 | 1.99 | 0.90 | 3.50 |
| xs.b | 0.41 | 0.25 | 0.46 | 0.25 | 0.46 | 0.18 | 0.06 | 0.42 |
| xs.c | - | - | 0.26 | 0.2 | 0.26 | 0.2 | - | - |
| xs.bp | - | - | 20.5 | 20.5 | 20.5 | 20.5 | - | - |
| dr.a | 2.88 | 7.81 | 2.62 | 1.78 | 2.70 | 1.90 | 4.90 | 2.97 |
| dr.b | -0.15 | -0.41 | -0.08 | -0.05 | -0.083 | -0.05 | -0.29 | -0.11 |
| dr.xs | 17.63 | 17.13 | 27.02 | 29 | 27.02 | 29 | 19.87 | 22 |
| dsl | 13 | 0.2 | 2.50 | 0.30 | 1.90 | 0.15 | 0.50 | 0.02 |
| L1 | 51.18 | 15.98 | 17.59 | 4.2 | 17.59 | 4.2 | 34.51 | 7.06 |

Table S2: Root mean square error (RMSE) and the coefficient of determination (R^2^) for the predictions of blade length, width and leaf area for maize, sorghum and pearl millet with either generic or specific parameters.

|  | Maize P1197 | | Sorghum Tx7000 | | Sorghum R931945_2_2 | | Pearl millet NPM-3 | |
| --- | --- | --- | --- | --- | --- | --- | --- | --- |
|  | RMSE | R^2^ | RMSE | R^2^ | RMSE | R^2^ | RMSE | R^2^ |
| Blade length generic | 87.41 | 0.900 | 94.09 | 0.924 | 127.28 | 0.923 | 121.48 | 0.647 |
| Blade length specific | 63.17 | 0.943 | 70.21 | 0.930 | 53.96 | 0.953 | 49.74 | 0.935 |
| Blade width generic | 11.70 | 0.980 | 8.56 | 0.951 | 15.02 | 0.920 | 13.46 | 0.931 |
| Blade width specific | 4.56 | 0.984 | 7.32 | 0.950 | 6.38 | 0.970 | 4.85 | 0.915 |
| Leaf area generic | 96.65 | 0.911 | 46.56 | 0.930 | 83.86 | 0.904 | 34.84 | 0.860 |
| Leaf area specific | 64.81 | 0.960 | 61.44 | 0.913 | 67.42 | 0.945 | 21.34 | 0.860 |
